# Cognitive impairments are independently associated with shorter survival in diffuse glioma patients

**DOI:** 10.1007/s00415-020-10303-w

**Published:** 2020-11-19

**Authors:** Emma van Kessel, Irene M. C. Huenges Wajer, Carla Ruis, Tatjana Seute, Susanne Fonville, Filip Y. F. L. De Vos, Joost J. C. Verhoeff, Pierre A. Robe, Martine J. E. van Zandvoort, Tom J. Snijders

**Affiliations:** 1 Department of Neurology and Neurosurgery, University Medical Center Utrecht/UMC Utrecht Brain Center, G03.232, PO Box 85500, 3508 XC Utrecht, The Netherlands; 2 Experimental Psychology, Helmholtz Institute, Utrecht University, Heidelberglaan 1, 3584 CS Utrecht, The Netherlands; 3 Department of Medical Oncology, University Medical Center Utrecht/UMC Utrecht Brain Center, Q05.4.300, PO Box 85500, 3508 XC Utrecht, The Netherlands; 4 Department of Radiation Oncology, University Medical Center Utrecht, HP Q 00.3.11, 3508 GA Utrecht, The Netherlands

**Keywords:** Cognition, Diffuse glioma, Survival, Etiologic, Prognosis

## Abstract

**Background:**

Diffuse gliomas (WHO grade II–IV) are progressive primary brain tumors with great variability in prognosis. Cognitive deficits are of important prognostic value for survival in diffuse gliomas. Until now, few studies focused on domain-specific neuropsychological assessment and rather used MMSE as a measure for cognitive functioning. Additionally, these studies did not take WHO 2016 diagnosis into account. We performed a retrospective cohort study with the aim to investigate the independent relationship between cognitive functioning and survival in treatment-naive patients undergoing awake surgery for a diffuse glioma.

**Methods:**

In patients undergoing awake craniotomy between 2010 and 2017, we performed pre-operative neuropsychological assessments in five cognitive domains, with special attention for the domains executive functioning and memory. We evaluated the independent relation between these domains and survival, in a Cox proportional hazards model that included state-of-the-art integrated histomolecular (‘layered’ or WHO-2016) classification of the gliomas and other known prognostic factors.

**Results:**

We included 197 patients. Cognitive impairments (*Z*-values ≦ − 2.0) were most frequent in the domains memory (18.3%) and executive functioning (25.9%). Impairments in executive functioning and memory were significantly correlated with survival, even after correcting for the possible confounders. Analyses with the domains language, psychomotor speed, and visuospatial functioning yielded no significant results. Extensive domain-specific neuropsychological assessment was more strongly correlated to survival than MMSE.

**Conclusion:**

Cognitive functioning is independently related to survival in diffuse glioma patients. Possible mechanisms underlying this relationship include the notion of cognitive functioning as a marker for diffuse infiltration of the tumor and the option that cognitive functioning and survival are determined by overlapping genetic pathways and biomarkers.

**Electronic supplementary material:**

The online version of this article (10.1007/s00415-020-10303-w) contains supplementary material, which is available to authorized users.

## Introduction

Diffuse gliomas (WHO grade II–IV) are progressive primary brain tumors with a variable, but generally poor prognosis, despite recent progress in treatment options. Until now, research yielded several important predictors of survival, including age, Karnofsky performance status, and tumor grade (and histomolecular classification) for both high and low-grade glioma. Additionally, several prognostic factors for specific grades of tumors were reported: for low-grade glioma, presence of neurologic deficits before surgery (regardless of epilepsy), pre-operative tumor-volume, and midline crossing. For high-grade glioma: *MGMT* promoter methylation status, extent of resection and MMSE (minimal mental state examination) score [1, 2]. These prognostic factors are important to personalize treatment and rehabilitation. Additionally, identification of certain molecular or cognitive prognostic markers can lead to new insights in the pathophysiological mechanisms of diffuse glioma.

Cognitive deficits occur in all different grades of glioma [3, 4]. Several studies already revealed that these deficits are significantly associated with survival in diffuse gliomas [5, 6]. However, as the focus of this previous research was prognostic rather than etiologic, important covariates that influence both cognitive functioning and survival may have biased this relationship [6]. Whether this relationship between cognitive functioning and survival is truly independent, after correction for all known possible confounders, is unknown.

Of note, in most research, the MMSE score was used as the objective cognitive measure. MMSE is a screening tool that provides a measure of global cognitive dysfunction and is developed to screen for Alzheimer’s disease [7–9]; Although it has a proven prognostic value, it is neither a sensitive score nor can it be used to identify problems in given cognitive domains [10]. More specific scores for cognitive functioning can possibly predict survival in diffuse glioma patients more precisely and may reveal a causal relation of cognition, or its subcomponents, with survival [5, 6, 11].

To our knowledge, research in this field has been focused mainly on HGG and no data have been published about cognition as a predictor of survival for diffuse gliomas based on the World Health Organization (WHO) 2016-classification of (Central Nervous System) CNS tumors. In this work, we performed a retrospective cohort study with the aim to confirm the independent relationship between cognitive functioning in treatment-naive patients with diffuse gliomas (of all different grades) and survival, and to discuss the potential mechanisms that underly this relationship. We studied five predefined cognitive domains, with special focus on the domains executive functioning and memory; based on the high prevalence of impairments in these domains in glioma patients [3–5], we hypothesize that deficits in executive functioning and memory are most strongly related to survival in patients with diffuse glioma. Extensive domain-specific neuropsychological testing is more sensitive to these changes in cerebral network organization than MMSE and we, therefore, hypothesized that domain-specific scores from the extensive neuropsychological assessment are more strongly associated with survival than MMSE.

## Materials and methods

### Design

We performed a single-center retrospective study in a cohort of treatment-naive diffuse glioma patients who underwent neuropsychological testing as part of their pre-operative work-up for awake brain surgery between 2010 and 2017 at the University Medical Center in Utrecht, The Netherlands (UMCU). In the study sample, we analyzed the correlation of NCF scores for five predefined cognitive domains: executive functioning; memory; psychomotor speed; language; and visuospatial functioning on the one hand, with survival on the other. In our analysis, we had special attention for the domain's executive functioning and memory. The predetermined test classification system for all domains is shown in Table 1.

**Table 1:** Neuropsychological tasks per domain

Attention and executive functioning
Wechsler Adult Intelligence Scale (WAIS) Digit Span Forward^a^ Trail Making Test (TMT) Switching ratio (TMTB/TMTA)^b^ Phonologic Fluency^c^ Stroop/Delis Kaplan Executive Function System (DKEFS) inhibition ratio^d^ Wechsler Adult Intelligence Scale (WAIS) Digit SpanBackward
Memory
RAVLT-Dutch Version immediate, delay, recognition^e^ Rey-Osterieth Complex Figure Test (ROCF) delay^f^ Semantic Fluency^g^
Visuospatial functioning
Judgment of Line Orientation (JULO)^h^ ROCF direct
Psychomotor Speed
Stroop/DKEFS I Stroop/DKEFS II TMTA
Language
Boston Naming Test^i^ Token Test^j^

^a^Wechsler Adult Intelligence Scale Third Edition Digit Span [WAIS-III] (WAIS-III Administration and scoring manual, 1997), Wechsler Adult Intelligence Scale Fourth Edition Digit Span [WAIS-IV] [12]

^b^Trail Making Test [TMT] [13]

^c^Phonologic Verbal Fluency Test [Lexical Fluency] [14, 15]

^d^Delis-Kaplan Executive Function System [DKEFS] [16]

^e^15 Words Test [15WT] [17]

^f^Rey-Osterieth Complex Figure Test [ROCF] [18, 19]

^g^Semantic Verbal Fluency Test [Semantic Fluency] [14]

^h^Judgment of Line Orientation [JULO] [20, 21]

^i^Boston Naming Task [BNT] [22]

^j^Token Test [TT] [23]

### Participants

Data were obtained between January, 2010 and December, 2017 from a database. Prior to awake surgery, patients underwent an elaborate preoperative neuropsychological test battery. The inclusion criteria for this study were the presence of a diffuse glioma according to the criteria of WHO 2016 and a minimum age of eighteen years. For tumors diagnosed before 2016, we used all available histological and molecular data from clinical practice to (re-)classify the tumor according to WHO 2016 criteria. Exclusion criteria were:

- any form of tumor-directed treatment—such as tumor reductive surgery, chemotherapy, radiotherapy—before neuropsychological assessment. Having undergone biopsy shortly before a planned resection was allowed.
- incomplete neuropsychological assessment (due to emergency surgery of tumors simply located in the motor strip, for instance).

The UMCU institutional ethical review board approved the study; informed consent was not obtained for this observational study on data that were obtained as part of routine clinical care.

### Neuropsychological tests

The neuropsychological instruments that were used as part of our routine clinical care are listed in Table 1. These tests are internationally widely used, standardized psychometric instruments for assessing neurocognitive deficits (however not specific for oncology patients) [24]. All tests have normative data based on control subjects, with specific reference values per age group and, when appropriate, educational level, and sex. So by making use of *z*-values we corrected for educational level.

Neuropsychological tests often tap into more than one cognitive domain and classification into cognitive domains often varies in the literature. We made use of a predetermined test classification in accordance with previous studies and literature (Table 1) [25–27]. Our main domains of interest were memory and executive functioning. The domain executive functioning included aspects of attention [28–30]. The neuropsychological evaluation was conducted shortly (1–7 days) before the awake brain tumor surgery by an experienced neuropsychologist. Each neuropsychological test was scored according to standardized scoring criteria. For normative comparisons, the unadjusted scores were transformed into *Z*-scores based on the mean and standard deviation of control subjects derived from published norm data.

### Data collection

All neuropsychological data were prospectively collected between 2010 and 2017 in a database. We further extracted data on patient characteristics (also non-prognostic variables) from the electronic patient file for all diffuse glioma patients undergoing awake surgery in this period. Data included sex, age at surgical resection, integrated (‘layered’) histomolecular diagnosis based on WHO 2016 classification, Karnofsky Performance Scale score (KPS), MMSE (which is not part of neuropsychological assessment in our clinic), pre-operative tumor volume, and neurologic deficits or epileptic seizures at presentation [2, 31]. Volumes were measured in 3D with the use of Osirix Lite (v. 9.5.2) on T2-/fluid-attenuated inversion recovery (FLAIR)-weighted MRI scans and the volume was defined as the whole area of hyperintensity. This represents the total lesion volume, including tumor infiltration and edema. Volumes were measured by a junior clinical scientist (EvK) and a neuro-oncological neurosurgeon. Since this parameter is independent of enhancement (and thereby grade) of the lesion, it forms a widely usable representation of the extent of brain volume that is potentially hampered in its function by the tumor in any way [32].

Survival time was defined as the period between first resective neurosurgery and the date of death from cancer or any other cause, or censored at the date of last follow-up (March 1, 2019).

### Statistical analyses

Analyses were performed with RStudio (v1.1.463). We measured NCF data at the individual patient-level, which means that we counted the number of *individual* patients with an impaired performance per domain. A patient was considered impaired in a given domain if the patient performed below − 2 SD on *any* of the administered (sub)tests within that domain, in accordance with previous studies and based on clinical practice [4].

We analyzed baseline characteristics with descriptive statistics (Table 2).

**Table 2:** Baseline characteristics

	*N* (%)
Overall	197
Sex (Female)	69 (35.0)
Age at first surgery (mean (SD))	51.7 (14.7)
Domain Memory impaired	36 (18.3)
Domain Executive functioning impaired	51 (25.9)
Karnofsky Performance Score (median [IQR])	90.00 [80.00,90.00]
Volume (cm^3)^ (median [IQR])	54.91 [23.15, 101.56]
WHO2016 classification	
II + III astro IDH-M	47 (23.9)
II + III oligo IDH 1p19q codeletion	43 (21.8)
II + III astro IDH WT	12 (6.1)
IV GBM IDH M	6 (3.0)
IV GBM IDH WT	89 (45.2)
Location (measured on T2 FLAIR)	
Left hemisphere Right hemisphere Both hemispheres Left frontal Left parietal Left temporal Left occipital Right frontal Right parietal Right temporal Right occipital	133 (67.5) 56 (28.4) 8 (4.1) 98 (50.3) 47 (24.1) 71 (36.4) 21 (10.8) 54 (27.7) 34 (17.4) 27 (13.8) 8 (4.1)
Neurologic deficits at presentation	142 (72.1)
MMSE-score pre-operative (mean (SD))	28.09 (2.66)
Epilepsy at presentation (%)	130 (66.0)
Censoring	106 (53.8)
Overall survival (months) (mean (SD))	37.56 (35.24)

Before performing survival analyses, we tested for multicollinearity between determinants by Pearson correlation coefficients and considered an *R* of > 0.4 as collinear.

In order to avoid bias due to missing data, we imputed missing values for *all* variables by means of multiple imputation, through the R-package “Hmisc” *impute() *function for random missing values.

For the main analysis, we first examined the crude (unadjusted) relation between both the five cognitive domains of interest on the one hand and survival on the other, with univariable Cox proportional hazard models. We then investigated the adjusted effect between each of the five domains and survival, by correcting for all known possible confounding variables in Cox-regression models with a significance level of 5% (Tables 3, 4) [1, 2, 31].

**Table 3:** Univariable Cox-regression analyses

Variable	Crude HR (95% CI)	*p *value
Cognitive domain		
Executive functioning and attention^*^	3.26 (2.13–5.00)	< 0.0001^***^
Memory^*^	3.81 (2.43–5.99)	< 0.0001^***^
Psychomotor speed^*^	2.75 (1.78–4.25)	< 0.0001^***^
Visuospatial functioning^*^	2.16 (1.32–3.54)	0.002^***^
Language^*^	2.23 (1.27–3.92)	0.005^***^
MMSE	0.86 (0.81–0.92)	< 0.0001^***^
WHO 2016 glioma classification		
II + III astro IDH-M	Reference	NA
II + III oligo IDH-M 1p19q codeletion	0.38 (0.12–1.21)	0.10
II + III astro IDH WT	2.79 (0.94–8.27)	0.06
IV GBM IDH-M	1.52 (0.33–6.97)	0.59
IV GBM IDH WT	9.83 (4.90–19.73)	< 0.0001^***^
Tumor volume in cm^3^	1.004 (1.001–1.007)	0.003^***^
Age at first surgery	1.07 (1.06–1.09)	< 0.0001^***^
Karnofsky Performance Score	0.97 (0.95–0.98)	< 0.0001^***^
Sex (female)	0.66 (0.42–1.05)	0.08
Neurologic deficits at presentation	1.14 (0.72–1.81)	0.58
Epileptic seizures at presentation	0.59 (0.39–0.89)	0.01^***^

*HR *Hazard radio, *WT *wild-type mutated

*HRs represent hazard for impairment in the domain of interest

^***^p-value < 0.05

**Table 4:** Multivariable cox-regression analyses; corrected for WHO2016 classification, age, sex, Karnofsky Performance Score, Neurologic deficits, Epilepsy at presentation and pre-operative Tumor Volume

Model	Cognitive domain	AIC	Loglikelihood
1	Executive functioning	752.23	− 365.12 (*df* = 11)
1b	*Executive functioning, with interaction term included*	748.01	− 360.00 (*df* = 14)
2	Memory	751.90	− 364.95(*df* = 11)
2b	*Memory, with interaction term included*	753.87	− 362.94 (*df* = 14)
3	Psychomotor speed	761.35	− 369.68 (*df* = 11)
3b	*Psychomotor speed, with interaction term included*	764.98	− 368.49 (*df* = 14)
4	Visuospatial functioning	762.95	− 370.47 (*df* = 11)
4b	*Visuospatial functioning, with interaction term included*	766.59	− 369.29 (*df* = 14)
5	Language	761.06	− 369.53 (*df* = 11)
5b	*Language, with interaction term included*	762.95	− 367.48 (*df* = 14)
6	MMSE-score pre-operatief	757.10	− 367.55 (*df* = 11)

*Df *degrees of freedom, *AIC *akaike information criterium

We only included variables which potentially confound the relation between cognitive domain and survival. To act as a confounder, factors have to be associated with the determinant as well as the outcome. Consequently, we did not correct for applied treatment, the extent of resection, and location of the tumor. Applied treatment and extent of resection are established prognostic factors, but they cannot be of influence on baseline cognitive functioning, since surgery and further treatment occur post-baseline; as such, treatment and extent of resection cannot be confounders in this analysis. Location is not an established prognostic factor in literature, other than eloquently located tumors. All patients (with and without cognitive deficits) in our cohort had glioma in or near eloquent areas, as this location was the main clinical criterion to perform the awake operation, so there was no variation in an eloquent location within this cohort to correct for.

Since the various glioma subtypes differ greatly in their biological behavior as well as their prognosis, it is possible that the effect of cognition—and other determinants—on survival also differs between WHO 2016 glioma subtypes. To include this possibility in our Cox regression models, we extended the models with interaction terms to test for effect modification by WHO 2016 classification.

Since the Cox model assumes that survival curves of 2 strata follow hazard functions that are proportional over time, this proportional hazards (PH) assumption was checked for executive functioning, memory, and MMSE with log-minus-log plots.

To further test our hypothesis about the predictive value of MMSE, we first examined the adjusted hazard ratio by correcting for the same confounders as in the abovementioned models with cognitive domains. To compare model performance directly, we then compared the goodness of fit between the model that includes MMSE with and the models that include cognitive domain scores, specifically executive functioning and memory, by means of log-likelihood ratio tests.

## Results

### Clinical characteristics

In total 197 eligible patients underwent awake surgery between 2010 and 2017. Descriptive characteristics are presented in Table 2. Thirty-five percent of patients had a missing pre-operative MMSE-score. For the domains executive functioning and memory, only 2.0% of data was missing. All other variables had missing values between 1 and 2%, except WHO-2016 classification (11%) and KPS (5%). WHO 2016 classification and age were collinear with a correlation coefficient of 0.51. Because both variables are important potential confounders of the investigated relation, we decided to include both factors in the multivariable model.

### Neuropsychological data and survival

Cognitive impairments (*Z*-values ≦ − 2.0) were found for the domain memory in 18.3% of patients, and for executive functioning in 25.9%.

**Fig. 1:**
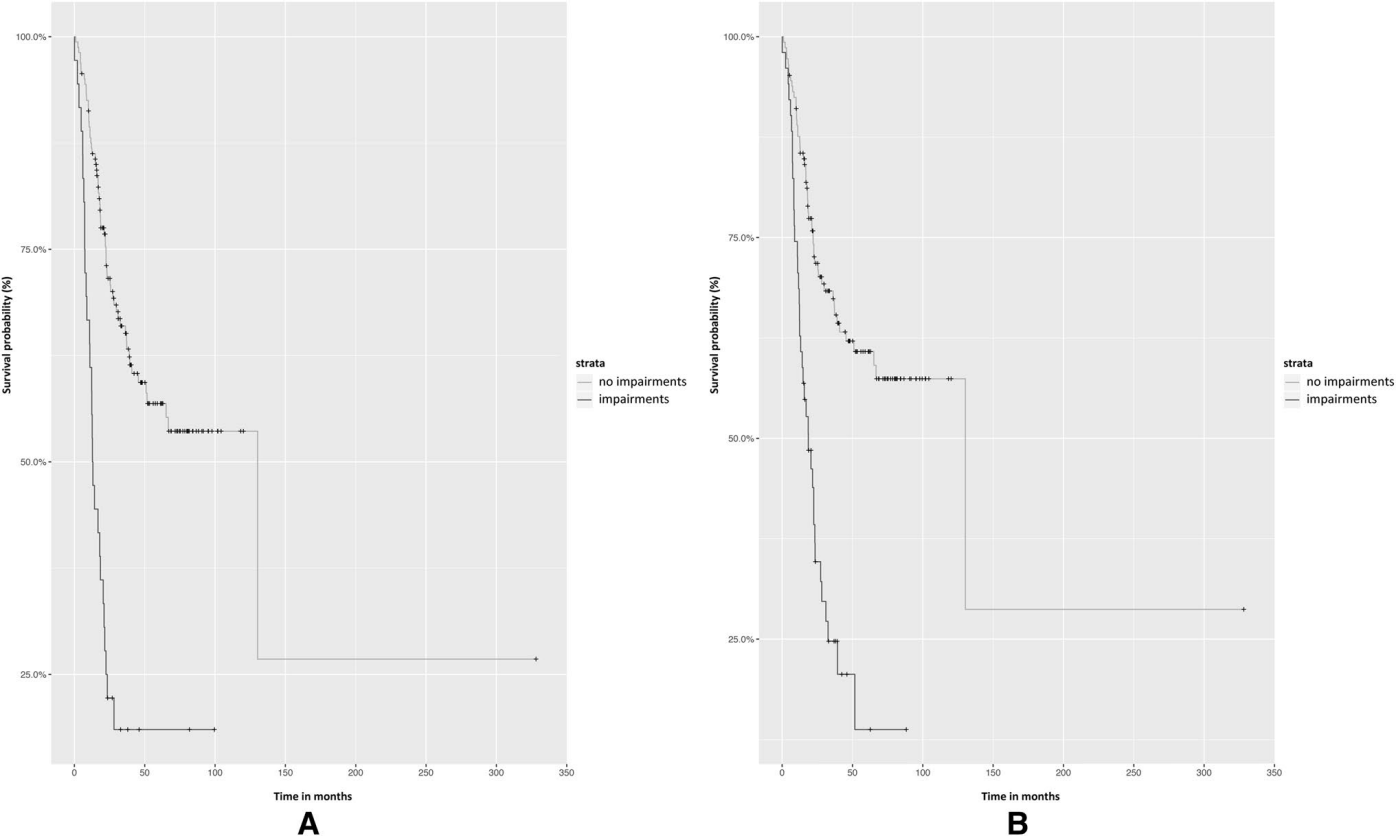
**a** Survival probability plot for the domain memory. **b** Survival probability plot for the domain executive functioning

The univariable survival analyses for all five cognitive domains, MMSE, and possible confounders resulted in crude hazard ratios, as shown in Table 3. The results of the goodness of fit (AIC’s and loglikelihood’s) for all cognitive domains and MMSE with and without interaction-term included in multivariable analyses are shown in Table 4. Cumulative survival curves are shown in Fig. 1. Supplementary Table 1 shows the adjusted hazard ratios of all determinants in multivariable cox-regression analyses. Interaction terms were only included if they improved the model.

Impairments in executive functioning {hazard ratio = 2.22 [Confidence interval (CI); 1.39–3.55]} and memory (hazard ratio = 2.43; 95% CI: 1.47–4.01) were significantly correlated with survival, even after correcting for the possible confounders: age, neurologic deficits, epileptic seizures, KPS, WHO-2016 classification, and pre-operative volume. While in the univariable model MMSE was significantly associated with survival, multivariable analysis showed worse fitting of our data with the MMSE model compared to executive functioning or memory models (in Table 4 AIC’s and loglikelihood’s are shown).

Because we found collinearity between age and WHO 2016 classification, we repeated cox regression analyses (to test for instability of the model) either without age or without WHO 2016 classification for both cognitive domains. This yielded very similar results, wherein the cognitive domains remained significant predictors in all variations of the model.

Effect modification by WHO 2016 classification seems to play a role in the relation between executive functioning and survival, as the multivariable Cox-regression model significantly improved (by means of loglikelihood) after adding the interaction term “WHO 2016 classification*Executive functioning impaired”. This means that the effect (hazard ratio) of the domain executive functioning on survival differs between the WHO 2016-subtypes of glioma. This effect modification does not apply to the domain memory.

Significant results for the domains executive functioning and memory hold true in subgroup analyses for grade II + III astrocytoma and oligodendroglioma IDH-M (*n* = 90) as well as II + III astrocytoma IDH WT, GBM IDH-M, and GBM IDH WT (*n* = 107).

We repeated the abovementioned analyses for the domains language, psychomotor speed, and visuospatial functioning. None of these domains (with *Z*-values ≦ − 2.0) showed significant results [language: HR = 1.67 (95% CI 0.88–3.18), *p* = 0.12; psychomotor speed: HR = 1.47 (95% CI 0.87–2.50), *p* = 0.0.15; visuospatial functioning: HR = 0.84 (95% CI 0.48–1.50), *p* = 0.53] Interaction terms did not improve these models in terms of loglikelihood. These results are shown in supplementary Table 1.3–1.5. Using a less conservative threshold for cognitive impairments of *Z* ≦ − 1.5 only resulted in significant findings for the domain psychomotor speed [HR = 1.79 (95% CI 1.10–2.91) *p* = 0.02].

## Discussion

Cognitive functioning is of great influence on the quality of life and also predicts survival in diffuse glioma patients. Impairments in the domains of executive functioning and memory are most prevalent (12). In this study, we focused on the independent relationship between five cognitive domains, with a focus on executive functioning and memory, and survival. We found that cognitive impairments in executive functioning and memory are negatively associated with survival in diffuse glioma patients. This association holds true, even when adjusted for well-established confounders including WHO 2016 classification and KPS. Since the various classes of diffuse gliomas differ in biological behavior, the relationship between cognitive functioning and survival may differ between WHO2016 classifications. Indeed, we found an interaction between the WHO 2016 classification and the relationship between executive functioning and survival, but no such interaction for the domain memory. These differences between both cognitive domains suggest that executive functioning and memory are both modified by different underlying histomolecular pathways and cerebral networks and therefore interact with histological features of the tumor in a different way. Even after incorporating these interaction terms in the model, the cognitive domains were still associated independently with survival.

Other cognitive domains showed no clear association with survival, with a possible exception for psychomotor speed, which only had predictive value when a threshold of ≦ − 1.5 was used.

As hypothesized, extensive domain-specific neuropsychological assessment is more strongly correlated to survival than MMSE. Incorporating other cognitive domains (language and visuospatial functioning) in our models did not show significant results.

Possible explanations for the revealed independent relationship between the cognitive domains executive functioning and memory and survival include the following;

- *Cognition is a marker for diffuse infiltration.* Most cognitive domains rely on widespread cerebral networks. Therefore, they are vulnerable to the effects of the structural nuance of the infiltrative tumor and metabolic changes in the tumor environment [33–35]. Speculatively, cognitive functioning may be hampered, before more structural changes occur and therefore can reflect the aggressiveness of the tumor in a more sensitive way than for instance MRI does. A possible explanation for the fact that analyses did not show significant results for the domains visuospatial functioning, psychomotor speed, and language is that impairments in cognitive functioning were more common in executive functioning and memory.
- *Cognition and survival share (genetic) risk-factors.* Another possible explanation is that neuronal signaling and survival are both influenced by common biomarkers and pathways. This might involve tumor biological changes (somatic mutations and alterations), other than included in the WHO 2016 classification [36, 37]. It may also involve germline alterations, as is illustrated by the study of Liu. et al. [37] who found different SNPs (single nucleotide polymorphism) to be significantly correlated with processing speed, executive functioning, and memory in diffuse glioma patients before anti-tumor treatment. Those were SNPs involved in inflammation, metabolism, and DNA repair pathways. Hypothetically, these SNPs can be involved in treatment response and disease progression either. Investigating the relationship between certain genetic factors and cognition was not part of this study, but will be in future research.
- *Cognition influences treatment decision making and treatment compliance.* Although cognition is not included in the criteria for post-operative treatment decision-making, cognition could have influenced the choice of therapy. Possibly, physicians consider patients with severe cognitive problems to be less eligible for more intensive therapies. Whether therapeutic decision-making mediates the relationship between cognitive functioning and survival, has to be investigated in further research. To our knowledge, no data on this topic has been published yet. Another possibility is patients with cognitive problems are less compliant to therapy or develop more complications, which in turn influences survival.

To our knowledge, this is the first study investigating the independent relationship between cognitive functioning and survival, corrected for WHO 2016 classification. Additionally, previous studies mainly focused on high-grade glioma and cognitive testing often consisted of MMSE or other cognitive screening tools [1, 5, 10]. Further major strengths of our study are the large sample size, the extensive standardized NCF testing prior to surgical resection, the conservative cut-off value of *Z*-values ≦ − 2.0 for cognitive impairments (which adds to the robustness of our findings), the completeness of data and the significant proportion of patients with tumor involvement of the right hemisphere.

Rather than measure cognitive changes postoperatively, pre-operative cognitive functioning was used to determine the impact of cognition on survival. Cognitive functioning at baseline represents the unbiased effects of the tumor on the underlying brain networks best, as cognitive functioning during follow-up can be influenced by surgical procedure and postoperative treatment as well. From a practical point of view, informing patients about their prognosis is most valuable in the earliest stages of the disease, when treatment choices have yet to be made. Additionally, we only adjusted for true confounders, because we were interested in the independent relationship between cognitive functioning and survival. For this reason, we decided not to correct for certain predictive factors such as applied treatment, the extent of resection, location of the tumor. These factors are commonly included in prognostic models, but are not true confounders: the majority of our patients (with and without our determinant of interest) had glioma in eloquent areas and both groups could have benefited from more advanced treatment strategies if they were treated recently. Additionally, location in itself (besides eloquent areas) is not a predictive factor for survival and therefore does not act as a confounder in the relationship between cognitive deficits and survival. Treatment and extent of resection probably act as intermediate factors instead of confounders; in other words, cognitive impairments can influence the choice of treatment and in this way affect survival-probability. Adjusting for intermediate factors is incorrect because it can bias the studied association. For this reason, treatment was not included in multivariable analyses.

Limitations of our study should also be mentioned. At our center, NCF was routinely performed in patients undergoing awake surgery, which thus conceives a selection bias. As reflected in Table 2, these patients may have different characteristics than those undergoing biopsy or standard resection. In addition, the percentage of LGG patients is higher in the group of awake surgery patients than in the total glioma population [4]. However, since we included all consecutive patients that underwent awake surgery, regardless of their cognitive performance or their outcome (survival), we feel that our analyses offer a valid description of the *relation* between cognitive performance and survival, without *selection bias* and without compromising the internal validity of our study. However, it is possible that this selection of patients has influenced the generalizability of our results.

Another factor that could have led to *selection bias* is the selective loss to follow up of patients who had insufficient neuropsychological data to perform analyses on. The reason for having insufficient data was often emergency surgery in case of rapid clinical decline (for full details see reference [8]), so this could have led to exclusion of patients with cognitive impairments and worse clinical performance and therefore we possibly underestimated the relation between cognitive functioning and survival.

Finally, we decided to group tasks on their conceptual background (‘domain’) to enhance power; analyses per task would add up to an undesirable number of analyses, and could potentially obscure findings for the overarching cognitive domain. The question of which cognitive concept (or domain) is best represented by a specific task is always complicated since intrinsically more than one concept is tapped in any task. However, neuropsychologists do share common ground in the categorization of tasks across domains [25–27].

Our findings support the hypothesis that cognitive functioning is independently related to survival in diffuse glioma patients and that extensive domain-specific neuropsychological assessment is more strongly correlated to survival than MMSE. When hypothesizing about possible mechanisms underlying this relationship, cognitive functioning may serve as a marker for diffuse infiltration of the tumor; alternatively, cognitive functioning and survival may be determined by overlapping genetic pathways and biomarkers. A deeper knowledge of the role of treatment as a mediator of the relationship between cognition and survival is needed, and additional studies on this relationship in glioma patients undergoing non-awake surgery are needed. Ultimately, (parts of) neuropsychological testing can be implemented in prognostic models for glioma patients.

## Availability of data and material (data transparency)

The data that support the findings of this study are available from the corresponding author, upon reasonable request.

## Electronic supplementary material

Below is the link to the electronic supplementary material.

Supplementary file1 (DOCX 28 KB)
