# Supplementary material for: Cognitive impairments are independently associated with shorter survival in diffuse glioma patients
Source: J Neurol. 2020 Nov 19;268(4):1434–42. doi: 10.1007/s00415-020-10303-w (PMC7990824; doi:10.1007/s00415-020-10303-w)
Supplement: Supplementary file 1 — Supplementary file1 (DOCX 28 KB) [file 415_2020_10303_MOESM1_ESM.docx]

**Supplementary material**

**Title:** Cognitive impairments are independently associated with shorter survival in diffuse glioma patients

**Journal name:** Journal of Neurology

**Author names:** Emma van Kessel MD^*^, Irene M.C. Huenges Wajer PhD, Carla Ruis PhD, Tatjana Seute MD PhD, Susanne Fonville MD PhD, Filip Y.F.L De Vos MD PhD, Joost J.C. Verhoeff MD PhD, Prof. Pierre A. Robe MD PhD**, Martine J.E. van Zandvoort PhD**, Tom J Snijders MD PhD**

**Corresponding author.* Address for correspondence: University Medical Center Utrecht Brain Center, Department of Neurology, internal address G03.232, PO Box 85500, 3508 XC Utrecht, The Netherlands. Tel+31 88 7574744; Fax +31 30 254 2100; e-mail: [e.vankessel-2@umcutrecht.nl](mailto:e.vankessel-2@umcutrecht.nl)

ORCID: 0000-0002-6356-436X

** these authors contributed equally to this work.

**Supplementary table 1.1:** Multivariable cox-regression analyses for the model with executive functioning and attention, with interaction term included. EF = Executive functioning & attention. * p≦ 0.05

| **Variable** | | **Adjusted HR (95% CI)** | **p-value** |
| --- | --- | --- | --- |
| **Executive functioning and attention (impaired)** | | 5.97 (1.53 - 23.32) | 0.010* |
| **Glioma WHO 2016 diagnosis** | |  |  |
| "II + III astro *IDH-M*" | | Reference | NA |
| "II + III oligo *IDH 1p19q codeletion*" | | 0.17 (0.02 - 1.44) | 0.105 |
| "II + III astro *IDH WT*" | | 4.27 (1.24 - 14.77) | 0.022* |
| "IV GBM *IDH M*" | | 1.22e-07 (0.00 – inf) | 0.996 |
| "IV GBM *IDH WT*" | | 8.66 (3.28 - 22.92) | <0.0001* |
|  |  |  |  |
| **Karnofsky Performance Score** | | 0.99 (0.97 - 1.01) | 0.3586 |
| **Tumor volume in cm^3^** | | 9.98e-01 (0.99- 1.001) | 0.1875 |
| **Age at first surgery** | | 1.05 (1.02 - 1.07) | <0.0001* |
| **Sex (female)** | | 8.36e-01 (0.51 -1.38) | 0.481 |
| **Neurologic deficits at presentation** | | 8.13e-01 (0.49 - 1.34) | 0.413 |
| **Epileptic seizures at presentation** | | 7.60e-01 (0.47-1.24) | 0.270 |
| **Interaction term: Executive functioning & attention * Glioma WHO 2016 diagnosis** | | | |
| EF impaired: II + III oligo *IDH 1p19q codeletion* | | 4.69 (0.34 - 65.52) | 0.251 |
| EF impaired : II + III astro *IDH WT* | | NA | NA |
| EF impaired : IV GBM *IDH M* | | 4.190e+06 (0.00 – inf) | 0.996 |
| EF impaired : IV GBM *IDH WT* | | 2.85e-01 (0.07 - 1.19) | 0.085 |

**Supplementary table 1.2:** Multivariable cox-regression analyses for the model with memory. * p≦ 0.05

| **Variable** | | **Adjusted HR (95% CI)** | **p-value** |
| --- | --- | --- | --- |
| **Memory impaired** | | 2.44 (1.48 – 4.01) | 0.00044* |
| **Glioma WHO 2016 diagnosis** | |  |  |
| "II + III astro *IDH-M*" | | Reference | NA |
| "II + III oligo *IDH 1p19q codeletion*" | | 0.39 (0.12 – 1.27) | 1.12 |
| "II + III astro *IDH WT*" | | 2.88 (0.96 – 8.64) | 0.06 |
| "IV GBM *IDH M*" | | 0.64 (0.13 – 3.20) | 0.59 |
| "IV GBM *IDH WT*" | | 5.20 (2.42 – 11.15) | <0.0001* |
|  |  |  |  |
| **Karnofsky Performance Score** | | 0.999 (0.976 – 1.011) | 0.466 |
| **Tumor volume in cm^3^** | | 0.998 (0.996 – 1.002) | 0.379 |
| **Age at first surgery** | | 1.05 (1.03 – 1.07) | <0.0001* |
| **Sex (female)** | | 0.96 (0.58 – 1.58_ | 0.873 |
| **Neurologic deficits at presentation** | | 0.72 (0.44 – 1.19) | 0.201 |
| **Epileptic seizures at presentation** | | 0.76 (0.47 – 1.24) | 0.273 |
|  | |  |  |

| **Variable** | | **Adjusted HR (95% CI)** | **p-value** |
| --- | --- | --- | --- |
| **Psychomotor speed impaired** | | 1.47 (0.87 - 2.50) | 0.153 |
| **Glioma WHO 2016 diagnosis** | |  |  |
| "II + III astro *IDH-M*" | | Reference | NA |
| "II + III oligo *IDH 1p19q codeletion*" | | 0.41 (0.12 - 1.33) | 0.136 |
| "II + III astro *IDH WT*" | | 2.73 (0.91 - 8.18) | 0.073 |
| "IV GBM *IDH M*" | | 0.84 (0.17 - 4.11) | 0.829 |
| "IV GBM *IDH WT*" | | 5.12 (2.39 - 10.96) | <0.0001* |
|  |  |  |  |
| **Karnofsky Performance Score** | | 0.99 (0.98 - 1.01) | 0.668 |
| **Tumor volume in cm^3^** | | 0.999 (0.996 - 1.003) | 0.749 |
| **Age at first surgery** | | 1.05 (1.03 - 1.07) | <0.0001* |
| **Sex (female)** | | 0.82 (0.50 - 1.34) | 0.419 |
| **Neurologic deficits at presentation** | | 0.76 (0.46 - 1.24) | 0.270 |
| **Epileptic seizures at presentation** | | 0.81 (0.49 - 1.34) | 0.417 |
|  | |  |  |

**Supplementary table 1.3:** Multivariable cox-regression analyses for the model with psychomotor speed. * p≦ 0.05

**Supplementary table 1.4:** Multivariable cox-regression analyses for the model with visuospatial functioning. * p≦ 0.05

| **Variable** | | **Adjusted HR (95% CI)** | **p-value** |
| --- | --- | --- | --- |
| **Visuospatial functioning impaired** | | 0.84 (0.48 – 1.46) | 0.532 |
| **Glioma WHO 2016 diagnosis** | |  |  |
| "II + III astro *IDH-M*" | | Reference | NA |
| "II + III oligo *IDH 1p19q codeletion*" | | 0.42 (0.13 – 1.36) | 0.147 |
| "II + III astro *IDH WT*" | | 2.57 (0.86 – 7.70) | 0.091 |
| "IV GBM *IDH M*" | | 0.80 (0.16 – 3.96) | 0.787 |
| "IV GBM *IDH WT*" | | 5.64 (2.65-11.98) | <0.0001* |
|  |  |  |  |
| **Karnofsky Performance Score** | | 0.991 (0.98 – 1.009) | 0.354 |
| **Tumor volume in cm^3^** | | 0.999 (0.996 – 1.002) | 0.570 |
| **Age at first surgery** | | 1.05 (1.03 – 1.07) | <0.0001* |
| **Sex (female)** | | 0.80 (0.49 – 1.31) | 0.381 |
| **Neurologic deficits at presentation** | | 0.82 (0.51 – 1.36) | 0.456 |
| **Epileptic seizures at presentation** | | 0.76 (0.46 – 1.26) | 0.287 |
|  | |  |  |

**Supplementary table 1.5:** Multivariable cox-regression analyses for the model with language. * p≦ 0.05

| **Variable** | | **Adjusted HR (95% CI)** | **p-value** |
| --- | --- | --- | --- |
| **Language impaired** | | 1.67 (0.88 – 3.18) | 0.117 |
| **Glioma WHO 2016 diagnosis** | |  |  |
| "II + III astro *IDH-M*" | | Reference | NA |
| "II + III oligo *IDH 1p19q codeletion*" | | 0.43 (0.13 – 1.39) | 0.159 |
| "II + III astro *IDH WT*" | | 2.73 (0.91 – 8.12) | 0.073 |
| "IV GBM *IDH M*" | | 0.80 (0.16 – 3.95) | 0.787 |
| "IV GBM *IDH WT*" | | 5.56 (2.62 – 11.80) | <0.0001* |
|  |  |  |  |
| **Karnofsky Performance Score** | | 0.997 (0.98 – 1.02) | 0.704 |
| **Tumor volume in cm^3^** | | 0.99 (0.99 – 1.002) | 0.418 |
| **Age at first surgery** | | 1.05 (1.03 – 1.07) | <0.0001* |
| **Sex (female)** | | 0.75 (0.45 – 1.23) | 0.254 |
| **Neurologic deficits at presentation** | | 0.77 (0.48 – 1.26) | 0.304 |
| **Epileptic seizures at presentation** | | 0.73 (0.46 – 1.17) | 0.195 |
|  | |  |  |
